# A Study on the Thermal Degradation of an Acrylamide and 2-Acrylamido-2-Methylpropanesulfonic Acid Copolymer at High Temperatures

**DOI:** 10.3390/polym15122665

**Published:** 2023-06-13

**Authors:** Guicai Zhang, Yunling Ran, Ping Jiang, Haihua Pei

**Affiliations:** School of Petroleum Engineering, China University of Petroleum (East China), Qingdao 266580, China

**Keywords:** acrylamide and 2-acrylamido-2-methylpropanesulfonic acid copolymer, thermal stability, hydrolysis reaction, oxidative thermal degradation

## Abstract

As a temperature-resistant and salt-resistant polymer, acrylamide and 2-acrylamide-2-methylpropane sulfonic acid (abbreviated as AM-AMPS) copolymer is currently widely used in drilling, water control and oil production stabilization, enhanced oil recovery and other fields, but its stability under high temperature has been less studied. The degradation process of the AM-AMPS copolymer solution was studied by measuring viscosity, the degree of hydrolysis, and weight-average molecular weight at different temperatures and aging time. During the high-temperature aging process, the viscosity of the AM-AMPS copolymer saline solution first increases and then decreases. The combined action of the hydrolysis reaction and the oxidative thermal degradation leads to the change of the viscosity of the AM-AMPS copolymer saline solution. The hydrolysis reaction of the AM-AMPS copolymer mainly affects the structural viscosity of its saline solution through intramolecular and intermolecular electrostatic interactions, while the oxidative thermal degradation mainly reduces its molecular weight by breaking the main chain of the copolymer molecules, reducing the viscosity of the AM-AMPS copolymer saline solution. The content of AM and AMPS groups in the AM-AMPS copolymer solution at various temperatures and aging time was analyzed using liquid nuclear magnetic resonance carbon spectroscopy, demonstrating that the hydrolysis reaction rate constant of AM groups was significantly higher than that of AMPS groups. The contribution values of hydrolysis reaction and oxidative thermal degradation of the AM-AMPS copolymer at different aging time to viscosity were quantitatively calculated at temperatures ranging from 104.5 °C to 140 °C. It was determined that the higher the heat treatment temperature, the smaller the contribution of hydrolysis reaction to viscosity, while the bigger the contribution of oxidative thermal degradation to the viscosity of the AM-AMPS copolymer solution.

## 1. Introduction

Water-soluble polymers are important raw materials for oilfield development and are widely used in drilling fluid, completion fluid, polymer flooding, profile control and water plugging, acidification, fracturing, sand control and many other fields [1,2,3,4,5,6,7,8,9]. The structure of conventional water-soluble polymers is unstable under high-temperature and high-salt reservoir conditions, particularly the commonly used partially hydrolyzed polyacrylamide (HPAM) in oil fields, which may lead to high-temperature cross-linking, chain breaking, functional group destruction, precipitation, and viscosity reduction [10]. With the gradual development of oil and gas resources, the requirements for stable water-soluble polymers at high temperatures are increasing. The long-term stability of acrylamide and 2-acrylamide-2-methylpropane sulfonic acid (AM-AMPS) copolymer in high-temperature and high-salinity brines is better than that of HPAM [11], so the AM-AMPS copolymer is becoming increasingly favored by oilfield developers.

As the polymers are mostly injected into the formation as a solution [12], the thermal stability of the AM-AMPS copolymer in saline solution directly affects its application. At present, there are few studies on the thermal stability of the AM-AMPS copolymer in brine solution, and the majority of scientists are primarily interested in HPAM polymer. Many scientists, such as Audibert et al., have shown that the degradation of HPAM polymer is the fundamental reason for the viscosity change in its saline solution after heat treatment [13,14,15,16]. Shin et al. measured the thermal degradation of a kind of HPAM in distilled water. Although viscosity data at different temperatures and aging time were measured in the experiment, the results had no value for comparison because the highest temperature of the experiment was 60 °C and the longest aging time was 24 h [17]. Ma et al. studied the degradation mechanism of acrylamide polymers and revealed the degradation mechanism of side groups (amide and carboxyl groups) [18]. Fu et al. used thermo-gravimetric analysis and infrared spectrometry (TG-IR) and thermo-gravimetric and mass spectrometry analysis (TG-MS) test methods in nitrogen to investigate the degradation process of an acrylamide polymer. The results demonstrated that the acrylamide polymer would produce small molecules such as carbon dioxide, acrylonitrile, and acetonitrile at temperatures higher than 315 °C [19]. Silva et al. compared the weight loss curves of HPAM and N-substituted alkyl derivatives of HPAM, and proved that the process from 250 °C to 330 °C was mainly the condensation between adjacent amide groups, deamination, and the formation of imide; from 330 °C to 420 °C, it was primarily a process of dehydrogenation and carbon dioxide formation [20,21]. Zhu et al. mentioned that the polymer containing trace peroxide impurities or trace oxygen in the solution could produce primary free radicals and trigger a chain automatic oxidation reaction, thus promoting the generation of free radicals in the polymer chain. The molecular weight of the polymer was decreased by the chain-breaking reaction [22]. 

Muller divided the degradation of polyacrylamide into chemical degradation and thermal degradation [14,15]. He found that the hydrolysis reaction and oxidative degradation of HPAM at high temperatures were the main factors affecting the thermal stability, but did not quantitatively characterize their contributions to the viscosity change of the copolymer solution. Sandengen et al. pointed out that a feasible method to study the thermal stability of the AM-AMPS copolymer was by using high temperature aging to accelerate a chemical reaction [23]. Some scientists studied the hydrolysis stability of the AM-AMPS copolymer, and observed that the hydrolysis reaction of AM was carried out with apparent first-order kinetics [11,23,24], and the hydrolysis reaction rate of AMPS groups was very slow at first, which increased after the hydrolysis of AM groups to a certain extent. Luo et al. prepared acrylic acid and 2-acrylamide 2-methylpropane sulfonic acid (AA/AMPS) copolymer and AM/AA/AMPS copolymer, and the high-temperature degradation experiment of the copolymers was designed according to the working conditions of the drilling fluid [25]. The two polymers were degraded at 200 °C, 220 °C, and 240 °C, and the polymers were analyzed using Fourier Transform Infrared Spectroscopy (FTIR), Nuclear Magnetic Resonance (^1^H NMR), X-ray Photoelectron Spectroscopy (XPS), Gel Permeation Chromatography (GPC), TG-IR, etc. The results showed that the thermal degradation behavior of acrylamide polymer solutions occurred mainly in the side groups that were not resistant to temperature, which showed a decrease in the content of methine in the main chain. 

In short, the evaluation and use of temperature-resistant and salt-tolerant polymers is severely hampered by the lack of research into the rheological properties of AM/AMPS copolymer saline solution at high temperatures and the imperfection of current thermal degradation evaluation methods, which seriously restricts the evaluation and application of temperature-resistant and salt-tolerant polymers. 

In this paper, the viscosity, degree of hydrolysis, and weight-average molecular weight of an AM-AMPS copolymer saline solution (AMPS content: 23.6%) at different temperatures and aging time were measured using a high-temperature aging test. The high-temperature resistance of the AM-AMPS copolymer was studied, and for the first time the contributions of the hydrolysis reaction and oxidative thermal degradation of the copolymer to the viscosity change of the copolymer saline solution after high-temperature aging were quantitatively characterized. The results offer significant reference values for research on the thermal stability of the AM-AMPS copolymer.

## 2. Materials and Methods

### 2.1. Materials

The AM-AMPS copolymer (weight-average molecular weight of about 5 × 10^6^ g/mol) was prepared using an aqueous solution polymerization method at 10 °C, and the initiator was a redox system consisting of ammonium persulfate and sodium bisulfite. By setting the molar ratio of AM to AMPS in the polymerization reaction to 3:1, the AM/AMPS copolymer with AMPS monomer ratio of 25% could theoretically be obtained. A certain mass of 2-acrylamido-2-methylpropanesulfonic acid (AMPS) was weighed into an open reactor, and an appropriate amount of water was added to dissolve the AMPS. Use 10% sodium hydroxide solution as the pH value adjuster to adjust the pH value of the system to 8, and then add a certain quality of acrylamide. The ethylene diamine tetra acetic acid (EDTA) solution with a mass fraction of 200 mg·L^−1^ was prepared as a complexing agent. After 15 min of deoxygenization due to injecting nitrogen, the initiator with the mass fraction 125 mg·L^−1^ was added. Place it in a water bath of suitable temperature for 4 h to obtain a copolymer block under the protection of nitrogen atmosphere. 

NaCl, Ethylene diamine tetraacetic acid (EDTA) and HCl were purchased from Sinopharm Chemical Reagent Co., Ltd., Shanghai, China; deionized water was made in the laboratory. The constant temperature water tank and 501 type super constant temperature water bath were produced by Jincheng Guosheng Experimental Instrument Factory, Jiangsu, China. Constant temperature oven was produced by Jinghong Experimental Equipment Co., Ltd., Shanghai, China. 

Brookfield DV-II+ Pro Viscometer was produced by Brookfield Company of the United States. Electronic balance (BS224S, d = 0.0001) was produced by Germany Sartorius Company. DL50 automatic potentiometric titration instrument was provided by Mettler Toledo Company in the United States. The DAWN HELEOS II eighteen angles laser light scattering instrument was provided by Wyatt Technologies Corporation in the United States.

### 2.2. Methods

#### 2.2.1. Preparation of Copolymer Solution

The AM-AMPS copolymer was added into 10 g/L NaCl solution carefully to prepare a solution with a mass concentration of 5 g/L. The mixture was then stirred slowly for 24 h, allowed to keep still and swell for 72 h, and then stirred for an additional 2 h. 

#### 2.2.2. Determination of Copolymer Solution Viscosity

The copolymer samples were sealed with an alcohol blowtorch. For safety considerations, a series of samples were labelled and put into the aging tank. To balance the pressure in the ampoule bottles, some water was added in the aging tank and placed in a constant temperature oven for heat treatment. The oven temperatures were set at temperature ranges of 80 °C, 104.5 °C, 116 °C, 130 °C and 140 °C, respectively. The polymer samples were taken out at different aging time. Polymer solution viscosities were measured with a shear rate of 10 s^−1^ at 30 °C by using Brookfield DV-II+Pro viscometer. 

#### 2.2.3. Determination of the Degree of Hydrolysis of the Copolymer Solution

The degree of hydrolysis of copolymers was determined using the improved method proposed by Pang, X. et al. [26]. This method was based on the common acid-base titration method (national standard GB12005.6-89 [27]), and uses automatic potentiometric titration to determine the degree of hydrolysis. The process is mainly divided into the following steps: ① titrate the polymer solution with hydrochloric acid standard solution through DL50 automatic potentiometric titration titrator, and record the volume of hydrochloric acid consumed when the PH value is 3.0; ② measure and record the volume of hydrochloric acid consumed by distilled water of the same volume as the polymer solution; ③ calculate the degree of hydrolysis of the polyacrylamide sample by deducting the volume of hydrochloric acid consumed in distilled water. When the pH value of the polymer solution is 3.0, 99% of the carboxylate ions can be converted into carboxylic acids, so a pH value of 3.0 is used as the titration endpoint. The use of automatic potentiometric titration to assist in judging the end point can avoid personal judgment errors. Deducting the blank volume in titration can more accurately reflect the true value of the degree of hydrolysis of the sample, improving the accuracy of test results and testing accuracy.

#### 2.2.4. Determination of Weight-Average Molecular Weight of Copolymers

The weight-average molecular weight is the sum of the weight fractions of molecules with different molecular weights multiplied by their corresponding molecular weights. It can highlight the contribution of the part with the highest number of molecules to the average molecular weight, which better characterizes whether the copolymer has broken chains. Therefore, the weight average molecular weight was used for measurement in the research process. The weight-average molecular weight was determined with the static light scattering (SLS) method. The DAWN HELEOS II eighteen angles laser light scattering instrument was used. The light scattering measurement requires the solution to be in a dilute solution state, using 0.5 M NaCl filtered through a membrane provided by Milipore Company, Burlington, Massachusetts, USA (the pore size was 0.38 μm) as the solvent, and preparing experimental samples with concentrations of 0.10 g/L, 0.20 g/L, 0.30 g/L, 0.40 g/L, and 0.50 g/L, respectively. The copolymer solution samples were, respectively, filtered and dedusted with membranes. Adjust the instrument to a constant temperature of 25 °C. When the light scattering instrument is in a stable state, calibrate the instrument with toluene as the reference material and measure the instrument constant. The scattering angle was 10–180°; measure the scattering light intensity of the prepared copolymer sample solutions and solvents with different concentrations at different scattering angles. The Zimm chart was used for data processing and analysis, and the weight-average molecular weights were obtained.

#### 2.2.5. Determination of the Relative Content of Carboxylic Acid, AM, and AMPS Groups

The content of each monomer in the AM-AMPS copolymer was determined using liquid nuclear magnetic resonance spectroscopy of the polymer. The mass fraction of the copolymer solution used in the test was 10 g/L, and the content of heavy water was 20%. Set the testing temperature to 25 °C and use the DEPTQ (Distortionless Enhancement by Polarization Transfer Including the Detection of Quaternary Nuclei) pulse sequence with a cumulative count of 10,496 times. The resonance peaks with chemical shifts of 176 × 10^−6^ ppm–184.5 × 10^−6^ ppm on the nuclear magnetic resonance spectrum are attributed to amide group, AMPS, and carboxylate. By integrating the normalized area of the characteristic peaks in the ^13^C DEPTQ spectrum, the relative content of each functional group can be calculated.

## 3. Results and Discussion

### 3.1. Effect of Heat Treatment on the Rheological Properties of the AM-AMPS Copolymer Solution

**Figure 1:**
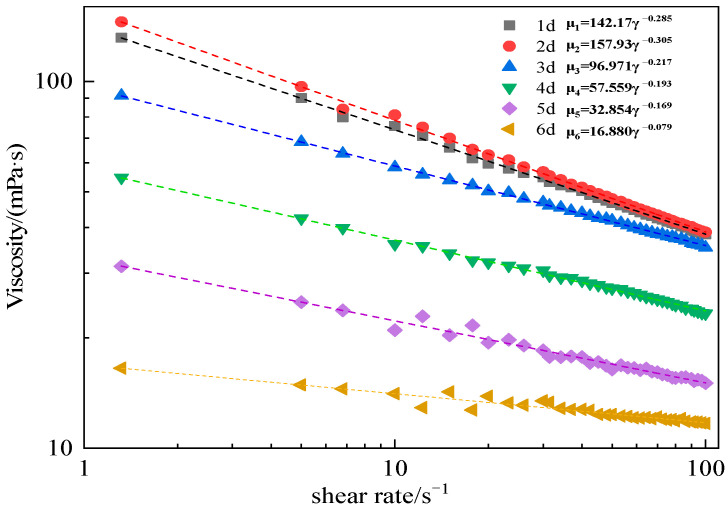
Rheological curves of the AM-AMPS copolymer solution at different aging times (116 °C).

By measuring the rheological curves of the AM-AMPS copolymer saline solution after different aging time under high temperature conditions, the impact of high-temperature aging on the measured viscosity of the AM-AMPS copolymer saline solution was studied. The rheological curves of the viscosity of the AM-AMPS copolymer solution (AMPS content is 23.6%) with shear rate at different aging time is shown in Figure 1, with a heat treatment temperature of 116 °C, and “d” in the figure represents “day of aging”.

It can be seen from Figure 1 that the relationship between the viscosity of the AM-AMPS copolymer solution and the shear rate conforms to a power law equation, as shown in the following formula:

(1) $$\mu =K\gamma^{n-1}$$

where *μ* is the measured viscosity of AM-AMPS copolymer, mPa·s; *K* is the consistency coefficient, mPa·s; *γ* is the shear rate, s^−1^; and *n* is the flow behavior index, which is dimensionless.

By comparing the consistency coefficients and flow behavior indices at different aging time, it can be seen that:

(1) Under high temperature heat treatment, the flow behavior index *n* in the rheological equation of the copolymer is less than 1, which indicates that the AM-AMPS copolymer saline solution is a typical pseudoplastic fluid;
(2) As the aging time lengthens, the consistency coefficient *K* declines, and the flow behavior index *n* progressively rises and approaches 1, so the AM-AMPS copolymer solution trends to a Newton fluid. These changes suggest that the thermal degradation of the AM-AMPS copolymer may take place at 116 °C.

### 3.2. Effect of Aging Time on the Viscosity of the AM-AMPS Copolymer Solution

**Figure 2:**
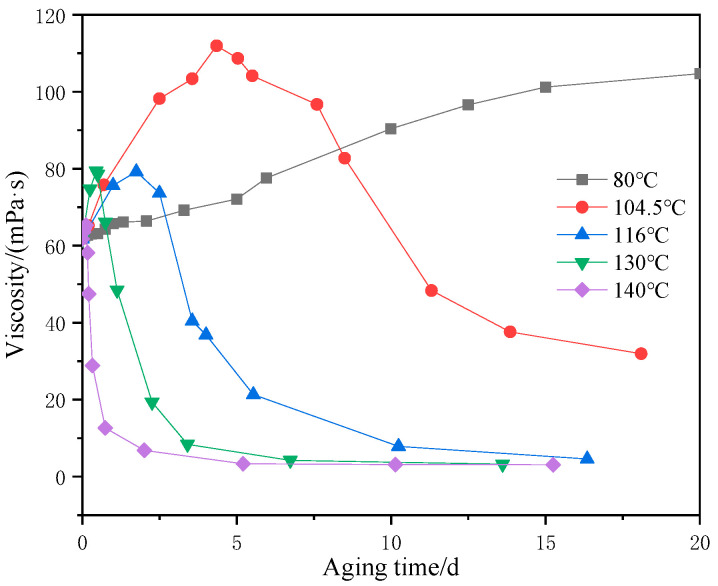
Viscosity of the AM-AMPS copolymer solution at different aging time.

To study the degradation law of the AM-AMPS copolymer solution, it is necessary to establish an indicator that describes the degradation. The measured viscosity at a shear rate of 10 s^−1^ is defined as “standard viscosity (*μ_b_*)”. The experiments below were all measured at 10 s^−1^, unless otherwise stated. The standard viscosity of the AM-AMPS copolymer solution was measured at different aging time for 80 °C, 104.5 °C, 116 °C, 130 °C, and 140 °C, as shown in Figure 2.

The results have shown that at the same temperature, the viscosity of the AM-AMPS copolymer solution initially increased to a peak value (μ_max_), and then rapidly decreased to a certain value, with the decline of rate becoming slower. This pattern was consistent with the results of Nurmi et al. [16].

**Figure 3:**
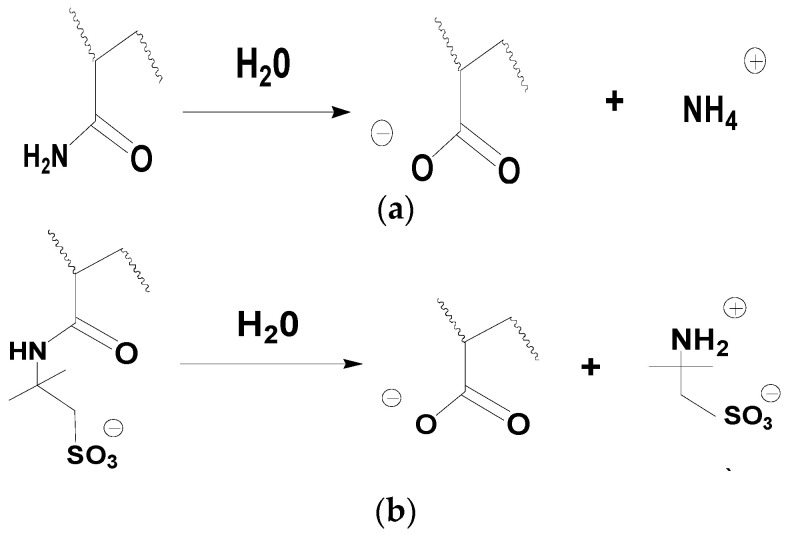
Hydrolysis reaction process of the AM-AMPS copolymer. (**a**) Hydrolysis reaction of the acrylamide group. (**b**) Hydrolysis reaction of the AMPS group.

Many scientists, such as Audibert, have pointed out that the degradation of polymers is the fundamental reason for viscosity changes in the solutions after high-temperature treatment [13,14,15,16]. According to Muller [14,15], there were two types of polyacrylamide degradation. The first one was caused by the hydrolysis reaction of polyacrylamide, which was called chemical degradation. That is to say, both AM and AMPS groups in the AM-AMPS copolymer could undergo hydrolysis reactions, as depicted in Figure 3. However, Nurmi did not clearly indicate the conditions and differences in hydrolysis reactions of different groups. The second one was the oxidative thermal degradation, which was induced by free radicals and mainly affected by oxygen, temperature, or light. 

### 3.3. Relationship between Viscosity and Temperature of the AM-AMPS Copolymer Solution

**Figure 4:**
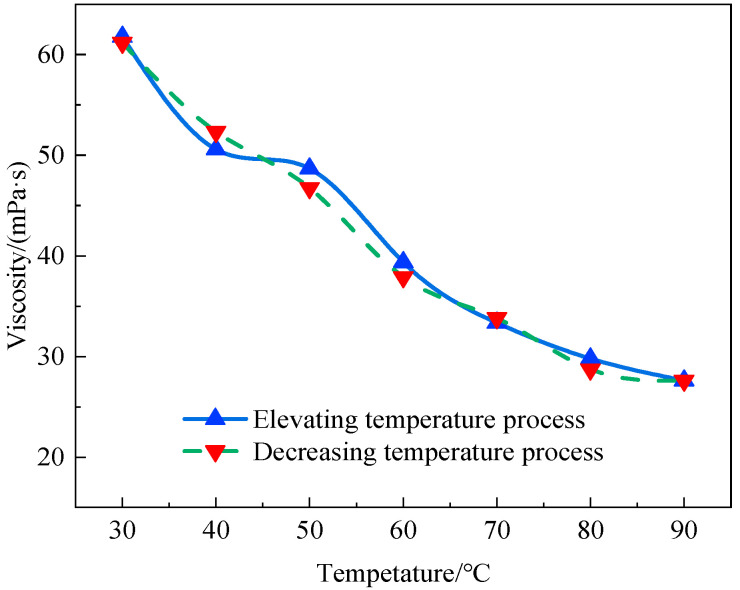
Viscosity changes with temperature in the AM-AMPS solution (10 s^−1^).

The viscosity of the AM-AMPS solution (AMPS content: 23.6%) was measured from 30 °C to 90 °C and back to 30 °C. The step temperature was set to 10 °C and the solution was thermostated at each temperature for 5 min. The viscosity value corresponding to the shear rate of 10 s^−1^ was measured using a Brookfield DV-II rheometer. As shown in Figure 4, the viscosity of the copolymer solution decreased with the increase in temperature and could return to the previous value, indicating that the temperature only affected the Newtonian viscosity and structural viscosity of the copolymer solutions during the test, and did not affect the copolymer molecules. The viscosity of the AM-AMPS solution decreased with the increase in temperature due to the increase in irregular thermal movement and the rotation ability of copolymer molecules in solution, and the entanglement points between polymers molecules were easier to open, resulting in a reduction of the entanglement density and the intermolecular force among copolymer molecules.

**Figure 5:**
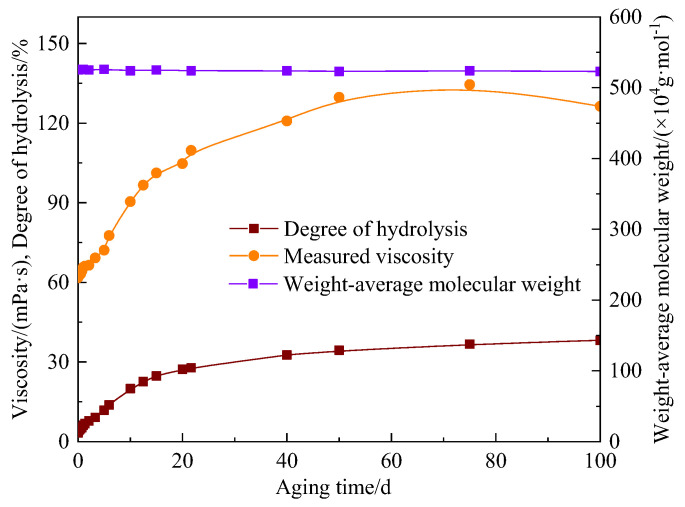
Changes in viscosity, the degree of hydrolysis and the weight-average molecular weight of the AM-AMPS copolymer solution at different aging time (80 °C).

In order to analyze the influence of the degree of hydrolysis on the viscosity, the viscosity, the degree of hydrolysis, and the weight-average molecular weight under different aging time at 80 °C were measured (Figure 5). The “0 d” in the figure represents the state of the AM-AMPS copolymer solution before being heated. 

It was evident that the viscosity of the solution firstly rose to its highest value (corresponding to the aging time of 75 days) with the increase in the degree of hydrolysis, and then decreased slightly. When the aging time was 100 days, the viscosity was still higher than the initial viscosity. The weight-average molecular weight of the AM-AMPS copolymer only decreased slightly with the aging time, indicating that the oxidative thermal degradation rate at 80 °C was extremely low and could be ignored, and that the hydrolysis reaction was the main reason for the viscosity change of the AM-AMPS copolymer solution. 

### 3.4. Hydrolysis Reaction of the AM-AMPS Copolymer Solution

#### 3.4.1. Hydrolysis Reaction Rate of AM and AMPS Groups

The content of AM and AMPS groups in the AM-AMPS copolymer solution at different aging time at 80 °C, 104.5 °C, 116 °C, 130 °C and 140 °C were analyzed using liquid nuclear magnetic resonance carbon spectroscopy (as shown in Table 1). 

It can be seen that the content of AM and AMPS groups in the copolymer decreased exponentially with the increase in aging time, indicating that the hydrolysis reaction of these groups conformed to the first-order reaction rate equation. Using the data in Table 1, the hydrolysis reaction rate constants of AM and AMPS groups at different temperatures can be calculated, as shown in Table 2. According to the hydrolysis reaction rate constants of AM and AMPS groups, AM groups are more likely to undergo hydrolysis reactions than AMPS groups.

#### 3.4.2. Effect of Temperature on the Degree of Hydrolysis 

**Figure 6:**
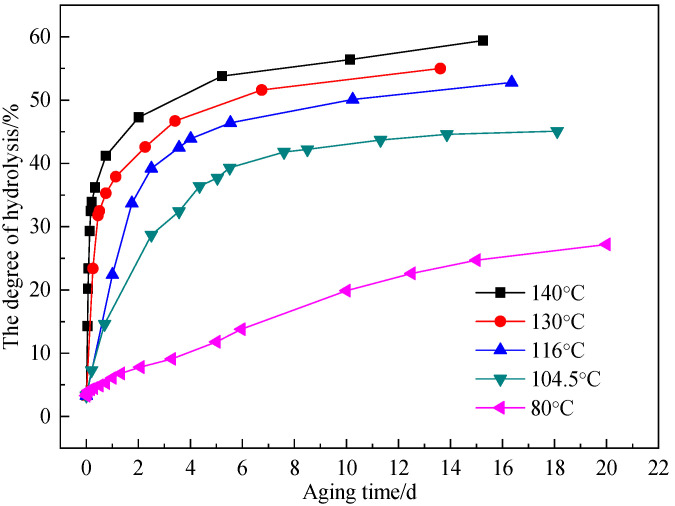
The degree of hydrolysis of the AM-AMPS copolymer solution at different aging time.

The degree of hydrolysis of the AM-AMPS copolymer solution was measured at different aging time from 80 °C to 140 °C (Figure 6). 

It was evident that as aging time rose, the degree of hydrolysis of the AM-AMPS copolymer increased before tending to a limit value and exhibiting a self-retardation effect. This conclusion was the same as that of Ryles’ [28]; in other words, the degree of hydrolysis tended to a limit value at a certain temperature, and the higher the temperature the greater the limit value of the degree of hydrolysis. The limit value of the degree of hydrolysis was about 45% at 104.5 °C, while it was approximately 60% at 140 °C.

#### 3.4.3. Relationship between the Degree of Hydrolysis and Measured Viscosity

**Figure 7:**
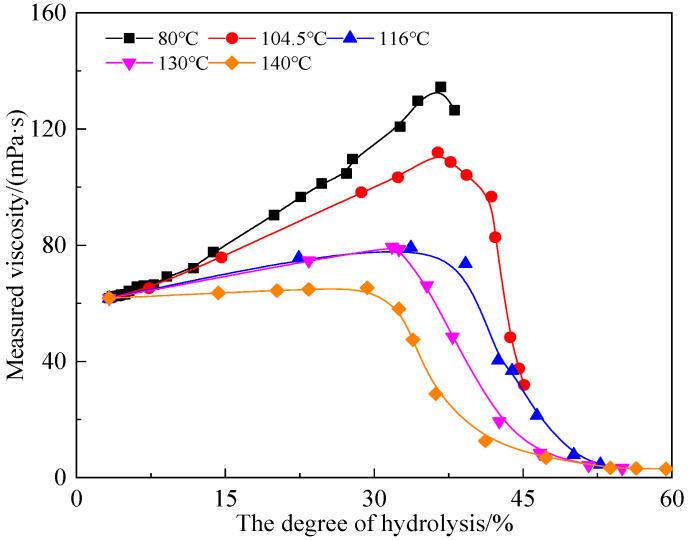
Relationship between measured viscosity and the degree of hydrolysis of the AM−AMPS copolymer solution at different temperatures.

The relationship between the measured viscosity and the degree of hydrolysis of the AM-AMPS copolymer solution was studied under different temperatures ranging from 80 °C to 140 °C (Figure 7). It is clear that the measured viscosity initially increased and then decreased with the increase in the degree of hydrolysis, and there was a peak viscosity (μ_max_). The higher the temperature, the smaller the μ_max_.

**Figure 8:**
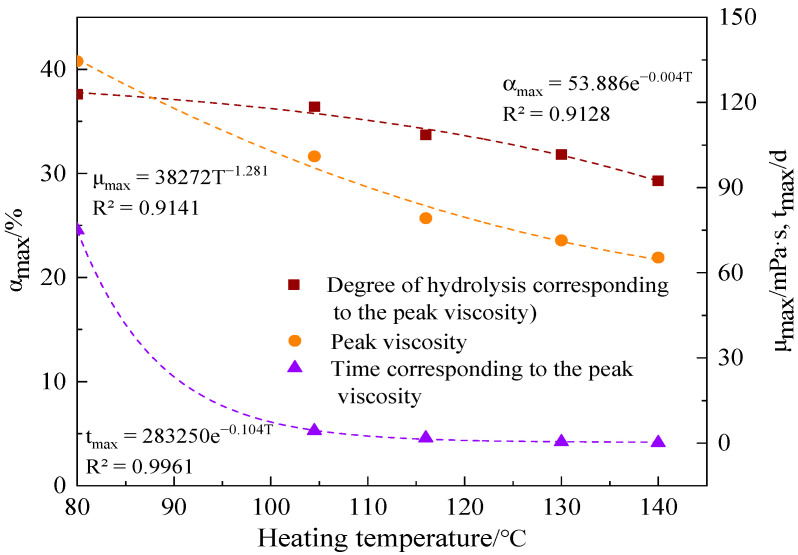
μ_max_, α_max_, and t_max_ of the AM-AMPS copolymer solution at different temperatures.

According to the above data, the peak viscosity (μ_max_) at different temperatures, the corresponding degree of hydrolysis (α_max_) and the aging time (t_max_) could be calculated. The results are shown in Table 3 and Figure 8. As can be observed, during the test μ_max_ decreased with the increase of temperature, while α_max_ and t_max_ decreased exponentially with the increase of temperature.

Contrary to Audibert’s conclusion [13], Table 3 demonstrates that the degree of hydrolysis corresponding to the peak viscosity (μ_max_) was not the same at different temperatures. Both the hydrolysis reaction rate and oxidative thermal degradation rate of the copolymer increased with the increase in temperature, and the viscosity of the copolymer increased due to hydrolysis reaction, while the viscosity decreased due to oxidative thermal degradation. Therefore, the optimal degree of hydrolysis will decrease if the thermal degradation change rate is greater than the hydrolysis reaction with temperature.

#### 3.4.4. The Mechanism of Viscosity Variation with Hydrolysis Reaction

**Figure 9:**
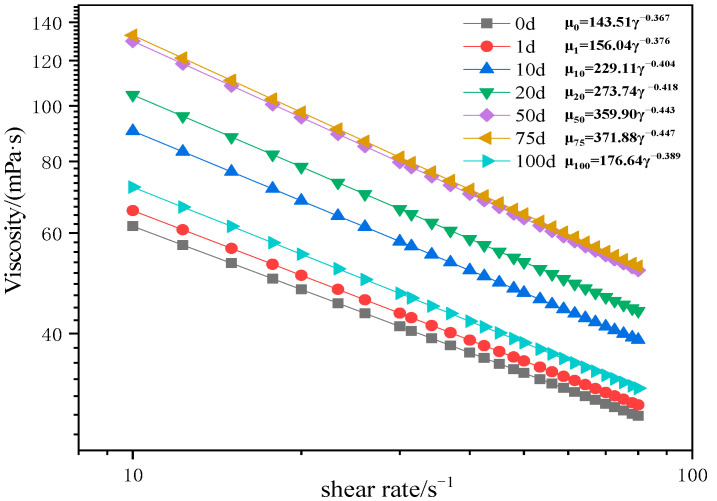
Rheological curves of the AM-AMPS copolymer solution (80 °C).

**Figure 10:**
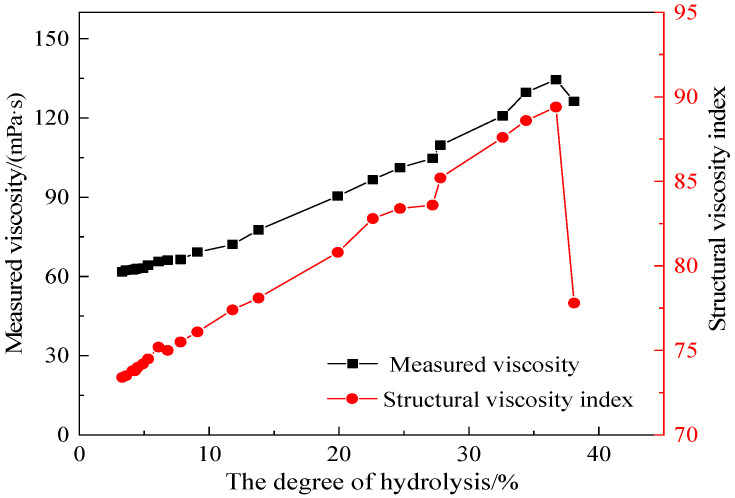
Changes in measured viscosity and structural viscosity index with the degree of hydrolysis.

In order to determine the reason for the changes in viscosity with the degree of hydrolysis, the rheological curve of the AM-AMPS copolymer saline solution at different aging time at 80 °C was measured (Figure 9). The structural viscosity index of the copolymer solution at different aging time (corresponding to different degrees of hydrolysis) was calculated (Figure 10) according to Formula (2) [29].

(2) $$∆\mu =\frac{dlg\mu }{dlg\gamma^{\frac{1}{2}}}\times 10^{2}$$

where *μ* is viscosity, mPa·s, and γ is shear rate, s^−1^. 

As seen in Figure 10, the viscosity of the AM-AMPS copolymer solution followed the same pattern as the structural viscosity index, suggesting that the change in the viscosity with the degree of hydrolysis was mainly caused by the change in the structural viscosity; that is, the structural viscosity changed due to the hydrolysis reaction. 

With the increase in the degree of hydrolysis of the AM-AMPS copolymer, the negative charge density and amount of copolymer molecules increased, leading to an increase in electrostatic repulsion among the chains in the molecule. Therefore, the hydrodynamic radius of the AM-AMPS copolymer in solution increased, which improved the contact and entanglement rate among molecules, thus increasing the structural viscosity. 

On the other hand, the electrostatic repulsion force among the copolymer molecules would also inhibit the entanglement of the intermolecular chains and reduce the structural viscosity. The hydrodynamic radius of the AM-AMPS copolymer had an upper limit value, while the inhibition of intermolecular chain entanglement always increased with the increase in electric charge, so the viscosity of the AM-AMPS copolymer solution increased initially and then decreased with the increase in the degree of hydrolysis.

### 3.5. The Oxidative Thermal Degradation of the AM-AMPS Copolymer Solution

In the temperature range of 73 °C to 120 °C, Nurmi et al. reported that the hydrolysis reaction of the copolymer was the factor that caused the viscosity change of the AM-AMPS copolymer solution [16], but the influence of oxidative thermal degradation was not considered. 

**Figure 11:**
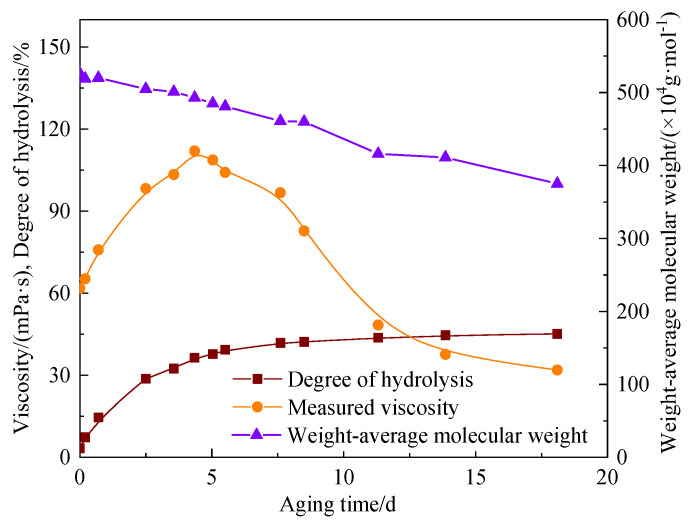
Changes in viscosity, the degree of hydrolysis and the weight-average molecular weight of the AM-AMPS copolymer solution at different aging time (104.5 °C).

In order to distinguish the effect of the hydrolysis reaction and oxidative thermal degradation of the AM-AMPS copolymer on the viscosity of saline solution, the relationship between the degree of hydrolysis, viscosity, and weight-average molecular weight was studied to determine whether oxidative thermal degradation occurred at 104.5–140 °C. The weight-average molecular weight of the AM-AMPS copolymer at different aging time at 104.5 °C was determined (Figure 11). Figure 11 shows that at 104.5 °C, the weight-average molecular weight decreased with the increase in aging time, which meant that the oxidative thermal degradation had occurred, leading to the breakage of the main chain of the molecules. 

Figure 5 shows that no significant change in the weight-average molecular weight of the AM-AMPS copolymer solution was observed after 100 days at 80 °C, so the oxidative thermal degradation could be ignored. The degree of hydrolysis had an exponential relationship with the viscosity of the AM-AMPS copolymer saline solution. Without considering the oxidative thermal degradation, the measured viscosity and degree of hydrolysis of the AM-AMPS copolymer saline solution followed the relationship expressed in Formula (3):

*μ_s_* = 56.391 e^0.0236*α*^ (3)

where *μ_s_* is the measured viscosity without considering oxidative thermal degradation, mPa·s, and *α* is the degree of hydrolysis, %.

The viscosity drop in the AM-AMPS copolymer saline solution caused by oxidative thermal degradation can be calculated as follows (taking 104.5 °C as an example).

Initially, the reference viscosity should be computed by disregarding oxidative thermal degradation (*μ*_s_) during the viscosity increase phase illustrated in Figure 3, utilizing Formula (4). Subsequently, the measured viscosity at the corresponding degree of hydrolysis (*μ*_t_) is subtracted from the reference viscosity (*μ*_s_) to derive the reduction value of oxidative thermal degradation viscosity (*μ*_r_). Thirdly, the retention percentage of oxidative thermal degradation viscosity (b) should be calculated using Formula (4) as presented in Table 4.

(4) $$b=100\times (1-\frac{\mu_{r}}{\mu_{s}})$$

**Figure 12:**
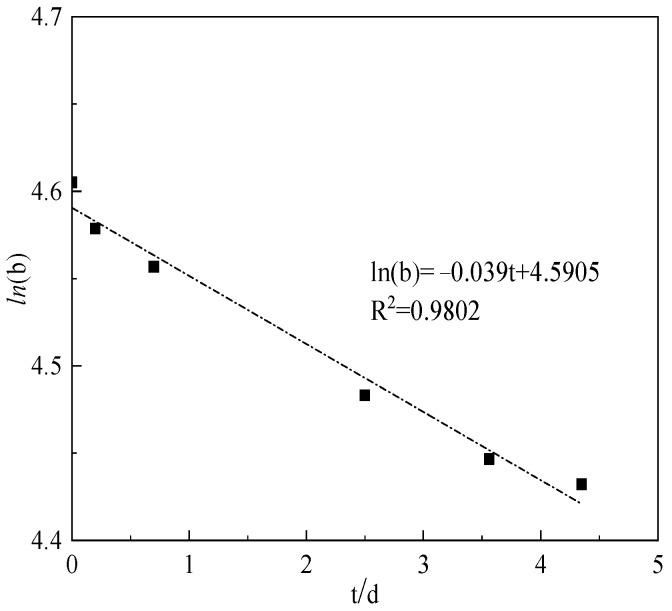
Curve of ln(b)-t of the AM-AMPS copolymer saline solution at 104.5 °C.

The present study plotted the natural logarithm of (b) against aging time, as illustrated in Figure 12. The observed linear relationship of ln(b)-t suggested that the oxidative thermal degradation adhered to the first-order reaction, with the linear slope representing the rate constant of oxidative thermal degradation ($K_{A}^{T}$) at 104.5 °C. This rate constant could quantitatively describe the variation in copolymer solution viscosity under different aging time. A higher $K_{A}^{T}$ value indicated a more severe oxidative thermal degradation of the AM-AMPS copolymer saline solution.

**Figure 13:**
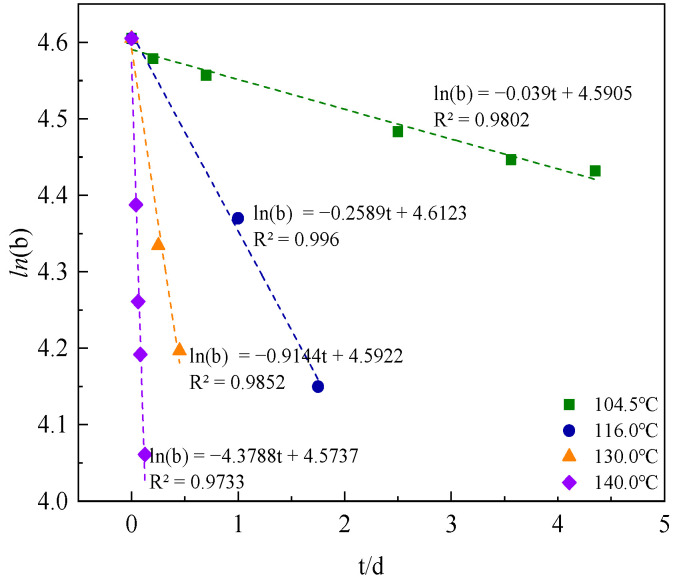
Curve of ln(b)-t of the AM-AMPS copolymer solution at different temperatures.

Utilizing the aforementioned methodology, the b value was computed for each temperature and aging duration. Subsequently, ln(b)-t was plotted (Figure 13) to determine the oxidative thermal degradation reaction rate ($K_{A}^{T}$) at varying temperatures (Table 5). 

The results demonstrated a positive correlation between the $K_{A}^{T}$ of the AM-AMPS copolymer and the experimental temperature, whereby an increase in the heat treatment temperature led to an increase in $K_{A}^{T}$. This observation suggested that an elevation in the aging temperature effectively exacerbated the oxidative thermal degradation of the AM-AMPS copolymer solution. This is because the main chain fracture of the copolymer molecules intensified as the temperature increased, resulting in a decrease in the weight-average molecular weight.

The initial viscosity can be defined as *μ*_0_, and the retention percentage *b_t_* of oxidation thermal degradation viscosity under different aging time calculated according to the equation of oxidation thermal degradation at 104.5 °C (*ln(b)* = −0.039 *t* + 4.5905). 

**Figure 14:**
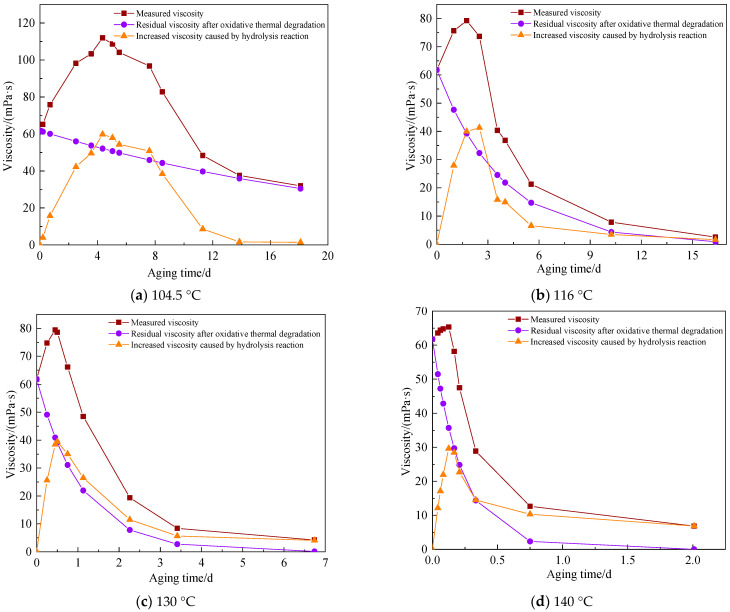
Contributions of hydrolysis reaction and oxidative thermal degradation to viscosity at different temperatures.

Formula (5) is the residual viscosity value after oxidative thermal degradation without the hydrolysis reaction ($\mu_{d}^{t}$), and Formula (6) is the increased viscosity value caused by the hydrolysis reaction ($∆\mu_{h}$); the results are shown in Figure 14a.

(5) $$\mu_{d}^{t}=\mu_{0}b_{t}$$

(6) $$∆\mu_{h}=\mu_{t}-\mu_{d}^{t}$$

It can be seen from Figure 14a that the measured viscosity of the AM-AMPS copolymer solution was the result of the joint action of copolymer hydrolysis reaction and oxidative thermal degradation, and the residual viscosity after thermal degradation gradually decreased with the aging time. As can be seen in Figure 14b through 14d, the contributions of hydrolysis reaction and oxidative thermal degradation to viscosity were calculated separately at 116 °C, 130 °C, and 140 °C. 

In summary, at temperatures ranging from 104.5 °C to 140 °C, the quantitative contributions of hydrolysis reaction and oxidative thermal degradation of the AM-AMPS copolymer at different aging time to viscosity were calculated. It was discovered that at the same aging time, the higher the heating temperature, the greater the decrease in viscosity due to oxidative thermal degradation. Furthermore, the higher the temperature, the shorter the time it took for the viscosity caused by the hydrolysis reaction to reach its maximum value. 

## 4. Conclusions

(1) The relationship between the measured viscosities of the AM/AMPS copolymer solution and shear rates conforms to a power law equation. As the high-temperature aging time increases, the consistency coefficient of the copolymer solution decreases and the flow behavior index increases, indicating that high temperature may cause the degradation of the AM-AMPS copolymer solution.
(2) With the increase of aging time, the viscosity of the AM-AMPS copolymer solution increases to a peak value before decreasing. The viscosity change of the AM-AMPS copolymer solution is the result of the joint action of the copolymer hydrolysis reaction and the oxidative thermal degradation. The hydrolysis reaction mainly affects the structural viscosity through intramolecular and intermolecular electrostatic interactions, while the oxidative thermal degradation mainly reduces its weight-average molecular weight by breaking the main chain of the copolymer molecules, thus reducing the viscosity of the AM-AMPS copolymer solution.
(3) As the high-temperature aging time increases, the degree of hydrolysis gradually approaches a limit value, and the higher the temperature, the greater the degree of the hydrolysis limit value. The content of AM and AMPS groups in AM-AMPS copolymer solution at different heat treatment temperatures and aging time has been analyzed using liquid nuclear magnetic resonance carbon spectroscopy. It was proves that the hydrolysis reaction of AM and AMPS groups conforms to the first-order reaction rate equation, and the hydrolysis rate constant of AM groups is significantly higher than that of AMPS groups.
(4) The contribution values of hydrolysis reaction and oxidative thermal degradation of the AM-AMPS copolymer at different aging time to viscosity were quantitatively calculated at temperatures ranging from 104.5 °C to 140 °C. It was determined that the higher the temperature, the smaller the contribution of the hydrolysis reaction to viscosity, while the bigger the contribution of the oxidative thermal degradation to the viscosity of the AM-AMPS copolymer solution.

## Figures and Tables

**Table 1:** The content changes of AM and AMPS groups at different high temperature aging time.

Temperature/°C	Aging Time/d	Content of AM/%	Content of AMPS/%	Degree of Hydrolysis/%
80	0	73.1	23.6	3.3
	1	70.3	23.6	6.1
	5	64.7	23.5	11.8
	10	56.6	23.5	19.9
	15	51.8	23.5	24.7
	20	49.4	23.4	27.2
104.5	0	73.1	23.6	3.3
	0.70	62.0	23.4	14.6
	2.50	48.2	23.1	28.7
	4.35	41.4	22.2	36.4
	7.60	36.2	22.0	41.8
	11.31	34.9	21.4	43.7
	18.10	34.8	20.1	45.1
116	0	73.1	23.6	3.3
	1	54.5	23.1	22.4
	2.50	38.4	22.4	39.2
	4.01	34.5	21.6	43.9
	10.24	29.8	20.1	50.1
	16.35	28.8	18.4	52.8
130	0	73.1	23.6	3.3
	0.50	44.4	23.1	32.5
	1.13	39.6	22.5	37.9
	3.41	32.6	20.7	46.7
	6.74	30.0	18.4	51.6
	13.61	28.8	16.2	55.0
140	0	73.1	23.6	3.3
	0.17	45.1	22.4	32.5
	0.33	42.7	21.1	36.2
	0.75	38.8	20.0	41.2
	2.01	34.3	18.4	47.3
	5.21	30.9	15.3	53.8
	15.25	29.2	11.4	59.4

**Table 2:** Hydrolysis rate constants of AM and AMPS groups at different temperatures.

Temperature/°C	k_AM_	k_AMPS_
80	2.20 × 10^−2^	4.0 × 10^−4^
104.5	1.08 × 10^−1^	9.0 × 10^−3^
116	2.11 × 10^−1^	1.6 × 10^−2^
130	2.81 × 10^−1^	3.0 × 10^−2^
140	4.76 × 10^−1^	5.3 × 10^−2^

**Table 3:** μ_max_, α_max_, and t_max_ of the AM-AMPS copolymer solution at different temperatures.

T, °C	μ_max_, mPa·s	α_max_, %	t_max_, d
80	134.5	37.6	75.00
104.5	112.0	36.4	4.35
116	79.2	33.7	1.75
130	79.4	31.8	0.45
140	65.3	29.3	0.12

**Table 4:** Parameters of the AM-AMPS copolymer saline solution at different aging time.

Aging Time t, d	Degree of Hydrolysis α, %	μ_t_ (10 s^−1^), mPa·s	μ_s_, mPa·s	μ_r_, mPa·s	b, %	ln(b)
0.00	3.30	61.76	61.76	0.00	100.00	4.61
0.20	7.30	65.23	66.99	1.76	97.37	4.58
0.70	14.60	75.83	79.59	3.76	95.28	4.56
2.50	28.70	98.24	111.00	12.76	88.50	4.48
3.56	32.40	103.37	121.13	17.76	85.34	4.45
4.35	36.40	111.96	133.12	21.16	84.10	4.43

**Table 5:** $K_{A}^{T}$ of the AM-AMPS copolymer solution at different temperatures.

Temperature, °C	$K_{A}^{T}$
104.5	0.039
116.0	0.259
130.0	0.914
140.0	4.379

## Data Availability

The data generated and analyzed during this study are available from the corresponding author upon reasonable request.
